# Transfusion of Blood

**Published:** 1871

**Authors:** Carl Proegler

**Affiliations:** Late Surgeon German Army, Aurora, Illinois


Article IV.—*Transfusion of Blood.* By Carl Proegler, M.D., late Surgeon German Army, Aurora, Illinois.

I have the pleasure to lay before the medical fraternity a few interesting cases of transfusion of blood, and I do it very reluctantly, knowing that our text books contain but very little. There are only twenty cases on record, as far as I know. The late Franco-German war has developed a good many striking features interesting for the surgeon only, not having been noticed during the late American war.

Tremendous battles such as have not been fought since the beginning of known history, and masses hurled together for certain destruction as not even the war era of the first Napoleon can point out, left some physical phenomena, as I would call them. Take only the deaths of the German army, 119,000 men, 3,000 officers, among them quite a good many surgeons, and any one may see that the fighting was done with desperation on either side.

The troops who came out alive from the bloody battles of

Rezanoible, Mary aux Chenes, Mars la Tour, and Gravelotte, experienced strange feelings, dizziness, deafness, nervous irritability, etc. But more so the French troops after routing the Commune.

After being discharged from the medical service of the German army, I wanted to see the other side of the picture, and only by strict adherence to the French and English language, I escaped detection as a German. The scenes after the fall of Paris beggar description—it was an embodied hell on earth. No discipline, no order, nothing. I saw wounded men dying by the dozen for want of attendance. Doctor Lefevre and several Russian gentlemen had a kind of provisory hospital near the Passage Saumon in the neighborhood of the Rue Richelieu, and here it was where I had occasion to try transfusion of blood. Most of the amputated died with secondary hemorrhage, probably produced by the nervous shock and excitement. I can relate only surgical cases, and having had four successful cases, I may without presumption point out, that to my own mind, in all cases where there is a great loss of blood, either produced by wounds, or rectal, pulmonary and uterine bleeding, transfusion ought to be resorted to as the dernier resort.

As transfusion of blood has been but very little practiced, I might be excused for pointing out the indications for transfusion in surgical cases.

1. Where amputation has been delayed on some account, with but little chance for the patient, but where the latter insists upon an operation.

2. Re-amputation or resection on account of pyemia or extreme hemorrhage, either on account of dangerous after-bleeding, general debility, or a hemorrhagic diathesis.

3. Extreme prostration on account of shock, and where life seems to be in imminent danger.

In cases of amputation, provided the patient was, up to the time of operation, a healthy man, his own blood might be taken, and instead of opening a vein in the arm, any of the branches of the saphena can be taken. The blood may be thrown in, in the already cut veins, care being taken to take a vein which has not too large a sheath. Future experience must point out more fully the correctness or otherwise of this view.

If you decide to transfuse blood into the arm, the median basilic is the best. The patient ought to be placed in a recumbent position, so that the blood may flow towards the heart, and then the operator dissects down to the vein, carefully dissecting loose the cellular tissue for about half an inch, and a double thread passed under it, so that the vein may be drawn out, when the operation commences. You may either cut a V-shaped piece out of the vein or cut the vein half through.

The blood when taken from the arm of a person ought to fall into a basin previously warmed with a temperature of about 100°, stirring it slowly with some glass reeds, care being taken not to create any air bubbles. Some authorities use phosphate of soda to prevent coagulation, but I have not used it; the even temperature is the main thing in my mind. After stirring, it ought to be strained through a perfectly clean muslin cloth. The instrument used is a Pravay syringe, made of glass, and holding three ounces, with an india rubber tube and a fine steel or silver nozzle cut like a pen. The amount to be injected varies according to circumstance but it never ought to exceed 16 ounces.

The vein is then put upon a probe, the nozzle carefully held and inserted and closely held by an assistant, care being taken that no air enters. It requires a little skill to do it, but will soon be learned on dogs, etc. I practiced first on cadavers, having an abundance of them.

Case 1. Leautie, French soldier, wounded in the streets of Paris; gunshot wound of the thigh; conservative surgery tried, but on account of deep-seated ulceration, about a week after admission amputation was performed. Operator, Dr. Le Ballier. Patient was of delicate health, but had never been sick previous to admission; nervous sanguineous temperament; pulse before operation, 95; countenance pale, and coming very slowly under the influence of chloroform. On account of his pulse, had to leave off anesthesia. Operation ended without any material changes, but patient lost a good deal of blood in spite of first class assistance in the person of Dr. Boucher, who compressed the femoralis. Pulse feeble, respiration hurried, all the symptoms of approaching collapse, and transfusion was speedily resorted to; used patient's own blood, was stirred with a glass reed, preventing as much as possible the formation of bubbles, and with the proper temperature, strained through a muslin cloth. I injected about four ounces; and let me say here, that where the blood is injected in the leg, the vein ought always, in cases of amputation, where there is a round and short surface, to be dissected carefully upwards, so that the nozzle of the syringe may be safely inserted, placing the leg high up and the patient low down.

The effect of the transfusion was, in this case, readily seen, and the symptoms became decidedly better. I used in this case altogether, eight ounces. The patient was kept in a recumbent position for a little while, carefully watching the pulse and the heart.

Patient was not removed to his bed until four hours after the operation, feeding him in the meantime with beef tea and wine, and producing artificial heat by warm bottles, etc. Patient made a good recovery. This case would not have stood the fearful loss of blood if transfusion of blood had not taken place.

Case 2. Morrel, French soldier, wounded before Paris by a ball penetrating the left lung between the third and fourth ribs. I have never seen a more hopeless case from the beginning; patient would lose blood every day, and discharged about an ounce of pus during the day from both sides. With the best of treatment, he grew worse. When I saw him he had a frightful attack of hemorrhage from the lungs, the third he had in the course of two days. Pulse 120, breathing hurried, clammy sweat, extremities cold, in fact, all the signs of speedy dissolution.

Ordered internally—sesquichl. of iron and brandy, but without any perceptible change. In consultation with Dr. Bagirsky, Le Vallier, etc., it was concluded to try infusion of blood; the blood was taken from a robust Frenchman. The median basilic was dissected and transfusion began; used with him nine ounces. The appearance after the introduction of the first three ounces was very marked; pulse perceptible and fuller; systolic murmur was heard, etc.

I placed him under the guardianship of a medical student, who watched him carefully for the next two weeks. The patient was doing finely when, through the negligence of the head steward, his room was cleaned, by which he caught cold, inducing double pneumonia, of which he died. It is evident that in this case life was prolonged.

Case 4. Farganier, French soldier, wounded at the tibia, showed signs of pyemia, and amputation was performed. Patient was of a cachectic tint; pulse, 90; heart's action weak, besides having a hunterian chancre. All of the surgeons were of the opinion that there was but little chance for his recovery. Used the blood of a healthy French peasant, a brother of the patient. Injected, after operation was over, and he gradually began to sink, six ounces in the vein of the leg. With the help of stimulants and proper after-treatment made a recovery.

Our profession has for its great aim the prolongation of life, and if I have contributed something to make death less fearful, or even prolonged life for a few seconds, I have been richly rewarded. For my colleagues it is now to consider these points, and I hope that future experience will reveal perhaps a better method than I could devise.

Dr. W. Betz, of Heilbronn, Germany, tried transfusion of blood successfully in a woman laboring of hemorrhages from the stomach and bowels. In spite of all care, he relates, that two very minute bubbles of air were forced into the veins; the entrance of air was instantly followed by profound collapse, but by the use of cold water and other restoratives the patient was soon revived. The woman ultimately recovered.

As already said, transfusion must not be put off too long, until death is, as it were, already sitting upon the lips. There have been used from one to two pounds, but such quantities produce so much systemic disturbance that it is better not to use more than 16; in the cases related I have not used over eight. I had not to resort in the same subject to transfusion again. The fatal cases died soon after. Any functional disturbances, as headache, etc., following transfusion, should be quieted with hydrate of chloral, morphia, etc.

